# The Modulation of Cholesterol Metabolism Is Involved in the Antiviral Effect of Nitazoxanide

**DOI:** 10.3390/idr13030060

**Published:** 2021-07-14

**Authors:** Claudio Fenizia, Salomè Valentina Ibba, Claudia Vanetti, Sergio Strizzi, Jean-François Rossignol, Mara Biasin, Daria Trabattoni, Mario Clerici

**Affiliations:** 1 Department of Pathophysiology and Transplantation, University of Milan, Via F. Sforza 35, 20122 Milan, Italy; claudio.fenizia@unimi.it (C.F.); claudia.vanetti@unimi.it (C.V.); 2 Department of Biomedical and Clinical Sciences, University of Milan, Via G.B. Grassi 74, 20157 Milan, Italy; sibba@lsuhsc.edu (S.V.I.); sergio.strizzi@guest.unimi.it (S.S.); mara.biasin@unimi.it (M.B.); daria.trabattoni@unimi.it (D.T.); 3 Romark Laboratories, L.C., 3000 Bayport Drive, Tampa, FL 33607, USA; jean-francois.rossignol@romark.co; 4 IRCCS Fondazione Don Carlo Gnocchi, Via A. Capecelatro 66, 20148 Milan, Italy

**Keywords:** nitazoxanide, thiazolide, cholesterol metabolism, antiviral, FLU, interferon

## Abstract

We previously investigated the role of Nitazoxanide (NTZ), a thiazolide endowed with antiviral and antiparasitic activity, in HIV-1 infection. NTZ treatment in primary isolated PBMCs was able to reduce HIV-1 infection in vitro by inducing the expression of a number of type-I interferon-stimulated genes. Among them, NTZ was able to induce cholesterol-25-hydroxylase (CH25H), which is involved in cholesterol metabolism. In the present study, we wanted to deepen our knowledge about the antiviral mechanism of action of NTZ. Indeed, by inducing CH25H, which catalyzes the formation of 25-hydroxycholesterol from cholesterol, NTZ treatment repressed cholesterol biosynthetic pathways and promoted cholesterol mobilization and efflux from the cell. Such effects were even more pronounced upon stimulation with FLU antigens in combination. It is already well known how lipid metabolism and virus replication are tightly interconnected; thus, it is not surprising that the antiviral immune response employs genes related to cholesterol metabolism. Indeed, NTZ was able to modulate cholesterol metabolism in vitro and, by doing so, enhance the antiviral response. These results give us the chance to speculate about the suitability of NTZ as adjuvant for induction of specific natural immunity. Moreover, the putative application of NTZ to alimentary-related diseases should be investigated.

## 1. Introduction

The antiviral role of thiazolides has been previously investigated. Nitazoxanide (Alinia^®^, NTZ), a compound initially used as an anti-parasitic agent, was shown to be effective against many DNA and RNA viruses, including Influenza virus, HCV and HBV [1,2,3,4,5,6,7,8,9,10]. Notably, NTZ was also shown to inhibit in vitro HIV-1 replication and to upregulate the expression of proteins that are involved in cholesterol metabolism and efflux, such as cholesterol-25-hydroxylase (CH25H) and liver X receptor (LXR) [11], as well as those encoding lipid-transport proteins, such as the ATP-binding cassettes A1 (ABCA1) and G1 (ABCG1), by which intracellular excess of cholesterol is transported to extracellular acceptors [11].

There is an increasing body of literature showing a tight connection between immune inflammatory processes and sterol metabolism, including cholesterol transport, storage and excretion [12,13]. Thus, interferons (IFNs), one of the most potent anti-viral signaling molecules, simultaneously trigger a broad response by upregulating hundreds of interferon-stimulated genes (ISGs) [14,15]; among such ISGs, CH25H was identified as a potent antiviral effector. In fact, viral infections themselves result in the rapid induction of CH25H and the consequent production of STAT1-dependent 25-hydroxy-cholesterol (25-HC), an oxysterol involved in a plethora of metabolic and signaling pathways, including cholesterol biosynthesis and intracellular homeostasis [15,16,17]. Interestingly, 25-HC inhibits the growth of a wide range of enveloped viruses by inducing structural changes in the cellular membrane, thus impairing viral entry at the virus-cell fusion step [15,16,17,18]. Recently, the very same resistance mechanism was demonstrated for Zika virus [19]. These data suggested that 25-HC can act as a potent antiviral mediator [20,21].

It has become clear that reprogramming of cellular metabolism has a direct effect on viral replication [22] and, in particular, many steps of the viral life-cycle involve and exploit lipids [23,24,25]. Thus, both enveloped and naked viruses interact with the phospholipidic membrane in order to enter the cell with a wide variety of mechanisms: (1) using specific carbohydrate displayed on membrane lipids as entry-receptor (Papilloma Virus—HPV); (2) hijacking specific lipid combination as entry co-factor (Influenza virus, Ebola virus); (3) binding to HDL/LDL receptors (Hepatitis C Virus—HCV, Vescicular Stomatitis Virus—WVSV); (4) targeting lipid-raft mediated endocytosis (Herpes Simplex Virus—HSV, Foot and Mouth Disease Virus, CytoMegalo Virus—CMV, Simian Virus 40); and (5) manipulating specific lipids in order to trigger viral envelope fusion with the cell membrane (Dengue Virus) [26,27,28,29,30]. Once inside the host cell, viruses can affect the lipid homeostasis and intracellular signaling in order to steer the cell metabolism towards their needs. Common cellular targets are FASN and SREBP (Dengue), 3-hydroxy-3-methyl-glutaryl-coenzyme A (HMG-CoA) reductase (West Nile Virus—WNV) and PI4K (HCV, HPV and CMV) [31,32]. In order to generate a productive infection, virions have to be efficiently assembled. Enveloped viruses go through the budding process, acquiring membranes that can be provided not only by the plasma membrane (HIV, VSV, Influenza virus), but also by the ER (Dengue, WNV) or by different organelles (HSV, poxvirus). Viruses can also produce proteins which specifically interact with the cell membrane (HIV) or alter the lipid composition and the curvature of the membrane (HCV) in order to predispose it for the budding process [33,34].

In the present work, as a proof of concept, we tested in an in vitro system whether the cholesterol metabolism is involved in the antiviral response against Influenza Virus (FLU) and whether thiazolides could, indeed, enhance such a response.

## 2. Materials and Methods

### 2.1. Reagents

NTZ was supplied by Romark Laboratories, L. C. (Tampa, FL, USA) and was suspended in DMSO (Sigma Aldrich, St. Louis, MO, USA) to obtain a 40 μg/mL solution and stored at 2–8 °C.

Stimulation with FLU antigens was performed with viruses kindly provided by Dr. Shearer, National Institutes of Health (NIH, Bethesda, MD, USA). Two live UV-inactivated influenza viruses (FLU) were used: an influenza A virus (A/Bangkok/RX73 and A/Puerto Rico/8/34 strains; 1:800) and the 1998–1999 formula of influenza virus vaccine (1:5000; Wyeth Laboratories Inc., Marietta, PA, USA). The influenza virus vaccine was an inactivated trivalent subunit formulation that contains the hemagglutinin antigens of influenza A H1N1, influenza A H3N1 and influenza B virus strains (each at 30 mg/mL). Such preparation of viral antigens was diluted 1:1000 in complete medium for FLU stimulated conditions.

### 2.2. PBMC Isolation and Culture

Whole blood was collected for the specific purpose of this study from 12 healthy volunteers by venipuncture in Vacutainer tubes containing EDTA (Becton Dickinson, Franklin Lakes, NJ, USA), and peripheral blood mononuclear cells (PBMCs) were separated on lymphocyte separation medium (Organon Teknica, PA, USA). All the procedures were carried out in accordance with the GLP guidelines adopted in our laboratory. Cells were then washed and re-suspended in complete medium. After viability assessment, cells were washed and re-suspended in RPMI complete medium and seeded simultaneously in the following conditions: unstimulated, unstimulated in the presence of NTZ (10 µg/mL), FLU-stimulated (1:1000) and FLU-stimulated in the presence of NTZ. PBMCs were collected for gene expression analyses at 4 h. In order to establish the suitable NTZ concentration, dose-responses experiments were performed and 10 µg/mL was identified, consistently with what was previously observed [11].

### 2.3. RNA Extraction, Retro-Transcription (RT) and Real-Time PCR Analysis

RNA analyses were performed as previously described [35,36]. Briefly, RNA was extracted from PBMCs by using the acid guanidium thiocyanate–phenol–chloroform method. One microgram of RNA was reverse transcribed into cDNA by random hexanucleotide primers, oligo dT and 200 U MMLV reverse transcriptase (Clontech, Foster City, CA, USA). cDNA quantification for IFNγ, CH25H, oxysterol binding protein (OSBP), LXR, ABCA1, Sp3, HMGCS1, acetyl-CoA acetyltransferase 2 (ACAT2), CD36 and macrophage scavenger receptor 1 (MSR1) (Bio-rad, Berkeley, CA, USA) was performed by real-time PCR (CFX96 connect, Bio-rad, Berkeley, CA, USA). Reactions were performed using a SYBR Green PCR mix (Bio-rad, Berkeley, CA, USA) and amplified according to the following thermal profile: initial denaturation (95 °C, 15 min) followed by 40 cycles of 15 s at 95 °C (denaturation) and 20 s at 60 °C (annealing) and 20 s at 72 °C (extension). Results were expressed as ΔΔCt and presented as ratio between the target gene and the glyceraldehyde 3-phosphate dehydrogenase (GAPDH) housekeeping mRNA.

### 2.4. Statistical Analysis

Comparisons between groups were performed to evaluate immunological differences. An unpaired t-test was performed for each variable. *p*-values were considered statistically significant when below 0.05 (for instance, * ≤ 0.05; ** ≤ 0.001; *** ≤ 0.0001). Data analysis was performed using the GraphPad Prism^®^ 5 software (GraphPad, San Diego, CA, USA).

## 3. Results

**Figure 1:**
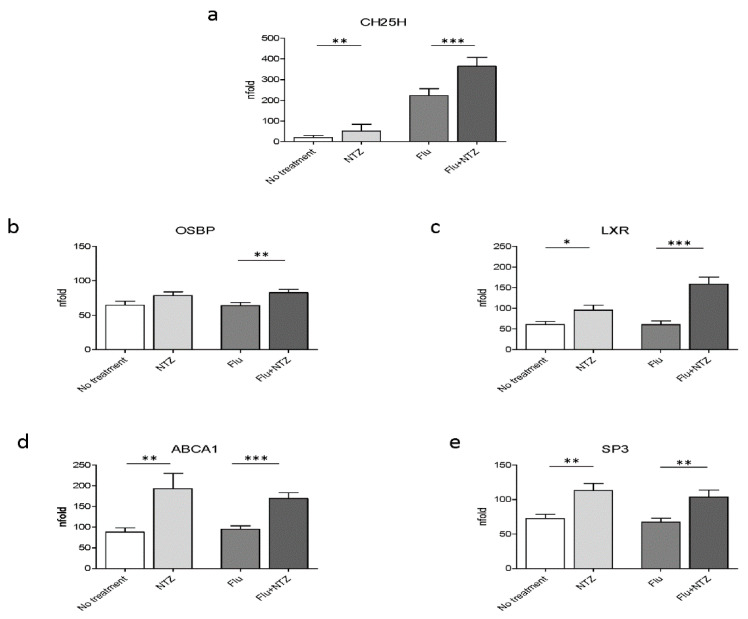
FLU and NTZ induce an immune response, affecting cholesterol intracellular homeostasis, by promoting cholesterol efflux. PBMCs from healthy donors (*n* = 12) were stimulated for 4 h with FLU antigens, NTZ or both in combination. CH25H was analyzed by Real Time PCR (**panel a**). mRNA level of OSBP (**panel b**), LXR (**panel c**), ABCA1 (**panel d**) and Sp3 (**panel e**) were analyzed by Real Time PCR. Mean values ± standard error are reported. The statistically significant differences are marked with one, two or three asterisks, which indicate a *p*-value equal or less than 0.05, 0.001 or 0.0001, respectively. Statistic was determined by t-test analyses.

Because IFNα and IFNβ are responsible for the induction of the transcription of ISGs, and in particular of CH25, a protein endowed with antiviral properties, we initially verified whether exposure of PBMCs to NTZ did upregulate CH25H transcription. Results shown in Figure 1a confirmed that CH25H expression was, indeed, significantly increased by NTZ. Thus, the addition of NTZ to PBMC cultivated in medium alone modestly increased CH25H transcription; CH25H transcription was much more potently activated in FLU-stimulated cells (*p* < 0.001 vs. FLU alone) (Figure 1a).

### 3.1. Cholesterol Homeostasis Genes

As a gene involved in cholesterol homeostasis, OSBP is upregulated upon NTZ treatment. Results showed that OSBP was upregulated in FLU-stimulated PBMCs (*p* < 0.001 vs. FLU alone) (Figure 1b). This is consistent with the observed CH25H increase and with its role in cholesterol homeostasis. 25HC exerts its antiviral role by activating LXR. LXR was upregulated as well, both in unstimulated (*p* < 0.05) and, more robustly, in FLU-stimulated PBMCs (*p* < 0.0001) (Figure 1c). The expression of ABCA1 was also increased by NTZ, both in cells cultured in medium alone (*p* < 0.001) and, more potently, in FLU-stimulated conditions (*p* < 0.0001) (Figure 1d). Sp3 expression was also upregulated as well by NTZ (Figure 1e). This was an unexpected finding given the observation that Sp3 is considered to be one of the negative regulators of the promoter of ABCA1 at the transcriptional level [37]. Although it is not fully characterized, upregulation of Sp3 could be part of a negative feedback mechanism [38], which is known to be finely tuned and tightly regulated by important post-transcriptional modifications.

### 3.2. Cholesterol Biosynthesis Genes

**Figure 2:**
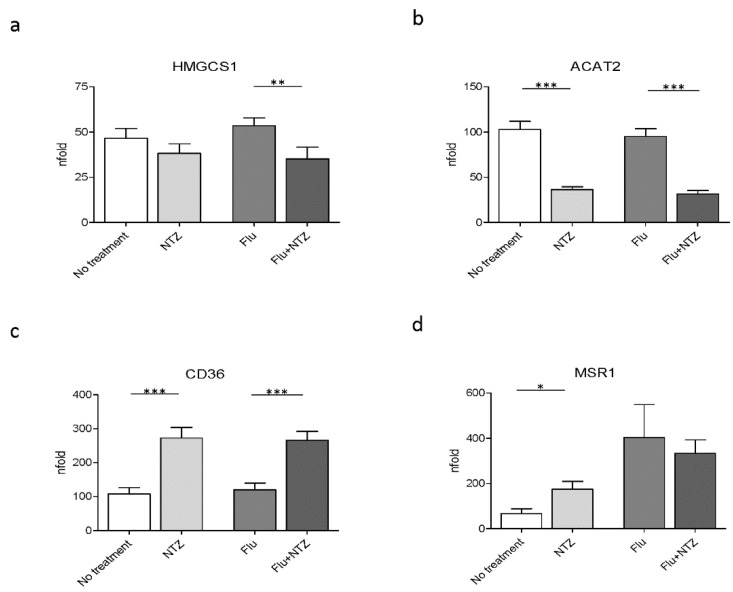
FLU and NTZ downregulate cholesterol biosynthesis, but induce scavenger receptors. PBMCs from healthy donors (*n* = 12) were stimulated for 4 h with FLU antigens, NTZ or both in combination. mRNA levels of the biosynthetic genes HMGCS1 (**panel a**) and ACAT1 (**panel b**), together with the scavenger receptors CD36 (**panel c**) and MSR1 (**panel d**), were analyzed by Real Time PCR. Mean values ± standard error are reported. The statistically significant differences are marked with one, two or three asterisks, which indicate a *p*-value equal or less than 0.05, 0.001 or 0.0001, respectively. Statistic was determined by t-test analyses.

We next verified whether, besides modulating genes involved in cholesterol homeostasis, genes that are part of cholesterol biosynthesis were also modulated by NTZ. The results showed that HMGCS1 was downregulated by NTZ, with a significant effect being observed in FLU-stimulated PBMCs (*p* < 0.001) (Figure 2a). NTZ also significantly downregulated ACAT2 expression both in unstimulated (*p* < 0.0001) and in FLU-stimulated cells (*p* < 0.0001) (Figure 2b).

### 3.3. Scavenger Receptor Genes

Scavenger receptors, such as CD36 and MSR1, are involved both in metabolism and immune inflammatory response, by participating in internalization of apoptotic cells, bacteria and viruses [39]. Their expression is regulated by many factors, including IFNs [39]. We observed that CD36 was upregulated by NTZ both in unstimulated (*p* < 0.0001) and in FLU-stimulated conditions (*p* < 0.0001) (Figure 2c). In contrast with these results, NTZ induced MSR1 upregulation in unstimulated cells alone (*p* < 0.05), but it had no significant effect on the expression of this gene upon FLU stimulation (Figure 2d).

## 4. Discussion

IFNs have a pivotal role both in innate and adaptive immune responses, as they regulate a plethora of processes, such as cell activation, cell differentiation, antigen presentation, migration, growth, proliferation and apoptosis. As a consequence of IFN production, hundreds of genes with immunoregulatory activity are induced; these genes are collectively named IFN stimulated genes (ISGs). Among them, genes encoding for proteins which regulate cholesterol metabolism are encompassed [40]. It has already been documented how lipid metabolism is tightly interconnected to virus life cycle and, thus, to antiviral responses. In particular, it was previously reported that the anti-HIV-1 activity of NTZ is associated with the modulation of genes involved in lipid and cholesterol metabolism, such as CH25H, LXR, PPARγ, ABCA1 and ABCG1 [11].

Cholesterol, on the other hand, is a component of every cell membrane, reducing its fluidity and permeability, and enhancing raft formation. It is also a precursor of steroid hormones, vitamin D and bile salts [40]. Its metabolism, comprising recirculation and distribution within the body, is finely tuned. Cholesterol can be absorbed with the diet or newly synthetized. Intracellular cholesterol concentration is constantly monitored. In fact, liver nuclear X receptors (LXR) act as oxysterol sensors of intracellular cholesterol homeostasis [37]. In case the concentration is excessive, new cholesterol biosynthesis is downregulated, while mobilization and efflux are promoted. Oxysterol-binding protein (OSBP) promotes the mobilization of lipids from ER to Golgi, together with the downregulation of newly synthetized cholesterol [41]. ATP-binding cassette A1 (ABCA1) modulates cholesterol efflux from cells by promoting HDL formation in an LXR-dependent manner [37]. In turn, specificity protein 3 (Sp3) is a negative transcriptional regulator of ABCA1, which counterbalances an excessive cholesterol efflux [42]. One of the first biosynthetic step of cholesterol is the formation of hydroxymethylglutaryl-CoA (HMGCoA) from Acetyl-CoA by HMGCoA synthase (HMGCS1) [40]. Interestingly, HMGCoA is the substrate of HMGCoA reductase (HMGCR), the target enzyme of statins. Once the cholesterol is synthetized, this molecule is either mobilized towards further metabolic processes or stored within cell membranes through esterification by acetyl-CoA acetyl-transferase (ACAT2), a process that regulates its bioavailability [40]. Cellular absorption of cholesterol is tightly regulated by specific scavenger receptors and transporters, such as CD36 and macrophage scavenger receptor 1 (MSR1) [39].

The observation that CH25H, an oxysterols-producing enzyme, is upregulated by thiazolides is particularly intriguing as it gives us the chance to further speculate about the anti-viral role of NTZ. In fact, the ability of NTZ to modulate lipid metabolism could exert a potent anti-viral activity by itself, as well as the already documented induction of interferons [11,43,44]. The antiviral activity of NTZ is not limited to HIV. In fact, it has been reported to be beneficial in in vitro models against a number of viruses, both in in vitro cellular models and in clinical trials. NTZ is effective against HBV (hepadnaviridae) [5] and multiple members of the flaviviridae, such as HCV [1,5,6], Zika virus [45], togaviridae, herpesviridae, Japanese Encephalitis Virus and others [8]. Moreover, not only it has been proven to be effective in vitro against influenza virus (FLU) [46,47,48,49], but it is also under investigation in clinical trials performed in patients with FLU infections [2,50].

**Figure 3:**
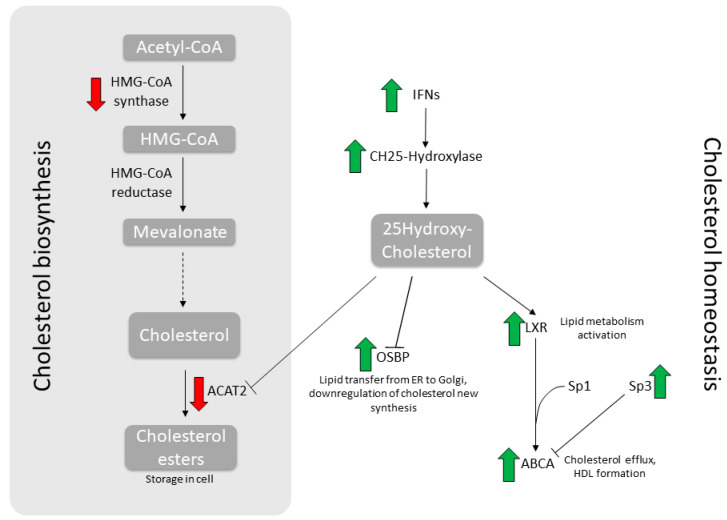
Synoptic summary of the main genes involved in cholesterol metabolism. Depicted here are the main metabolic steps in cholesterol biosynthesis (**left**) and homeostasis (**right**). Wide arrows indicate modulations induced by NTZ treatment.

Thus far, the connection between its antiviral activity and the ability to modulate cholesterol metabolism has been established for HIV only [11]. In the present work, we show that, as a proof of concept, both FLU antigens and NTZ treatment, singularly or combined for an even more pronounced effect, indeed trigger an antiviral response and modulate cholesterol metabolism. As a matter of fact, the homeostasis balance is skewed toward cholesterol mobilization and efflux, while the biosynthesis is inhibited, as summarized in Figure 3. Overall, cells decrease intracellular cholesterol level as antiviral mechanism, hampering viral trafficking and budding. Thus, this corroborates the rational for its employment as an adjuvant during antiviral treatment against FLU [2,3].

Cholesterol is a pharmacological therapeutic target of a number of diseases. In fact, according to the World Health Organization (WHO), among the top leading causes of death worldwide are alimentary-related diseases. In fact, either in high-income or in middle/low-income countries, the first cause of death is coronary heart disease (spanning from 17.1% to 14.6/10.8%, respectively). This is closely followed in the ranking by type 2 diabetes and hypertension in high-income countries only. Moreover, a number of other syndromes diffused among the population are considered major atherosclerosis risk factors, such as any cardiovascular disease (CVD), impaired glucose tolerance (IGT), dyslipidemia and inflammation. Immune cells, such as macrophages, are deeply involved in the atherogenic process, together with vascular endothelial cells, and they are important modulators of both lipid metabolism and immune responses. Accumulation of cholesterol-loaded macrophages in the arterial wall is the hallmark of the early atherosclerotic lesion. When the mechanism is overwhelmed, it leads to the development of foam cells and the fatty streak lesion. For this very reason, a number of molecules have been developed to specifically target cholesterol metabolism at different level [40], including genes involved in cholesterol uptake, homeostasis and biosynthesis, as reviewed by Charlton-Menys and Durrington [51]. Among them, statins are a well-known cholesterol biosynthesis inhibitor, by targeting HMGCR. Data herein suggest that thiazolides could have a role in the therapy of lipid dysmetabolism and related diseases. Indeed, they inhibit cholesterol biosynthesis and storage by downregulating HMGCS and ACAT2, respectively, and promote cholesterol efflux by upregulating LXR, OSBP and ABCA1. The putative beneficial or detrimental role of scavenger receptor upregulation (CD36 and MSR1), nevertheless, will need to be further investigated, as it is reported be part of the normal pro-inflammatory response [39], but it could also promote cholesterol uptake.

## Data Availability

The data presented in this study are available on request from the corresponding author.
